# Acute Aerobic Exercise Leads to Increased Plasma Levels of R- and S-β-Aminoisobutyric Acid in Humans

**DOI:** 10.3389/fphys.2019.01240

**Published:** 2019-09-25

**Authors:** Jan Stautemas, André B. P. Van Kuilenburg, Lida Stroomer, Fred Vaz, Laura Blancquaert, Filip B. D. Lefevere, Inge Everaert, Wim Derave

**Affiliations:** ^1^Department of Movement and Sports Sciences, Ghent University, Ghent, Belgium; ^2^Laboratory Genetic Metabolic Diseases, Amsterdam UMC, University of Amsterdam, Amsterdam, Netherlands

**Keywords:** myokine, exerkine, BAIBA, 3-amino-2-methylpropanoic acid, AGXT2

## Abstract

Recently, it was suggested that β-aminoisobutyric acid (BAIBA) is a myokine involved in browning of fat. However, there is no evidence for an acute effect of exercise supporting this statement and the metabolic distinct enantiomers of BAIBA were not taken into account. Concerning these enantiomers, there is at this point no consensus about resting concentrations of plasma R- and S-BAIBA. Additionally, a polymorphism of the alanine - glyoxylate aminotransferase 2 (AGXT2) gene (rs37369) is known to have a high impact on baseline levels of total BAIBA, but the effect on the enantiomers is unknown. Fifteen healthy recreationally active subjects, with different genotypes of rs37369, participated in a randomized crossover trial where they exercised for 1 h at 40% of P_peak_ or remained at rest. Plasma samples were analyzed for R- and S-BAIBA using dual column HPLC-fluorescence. The plasma concentration of baseline R-BAIBA was 67 times higher compared to S-BAIBA (1734 ± 821 vs. 29.3 ± 7.8 nM). Exercise induced a 13 and 20% increase in R-BAIBA and S-BAIBA, respectively. The AGXT2 rs37369 genotype strongly affected baseline levels of R-BAIBA, but did not have an impact on baseline S-BAIBA. We demonstrate that BAIBA should not be treated as one molecule, given (1) the markedly uneven distribution of its enantiomers in human plasma favoring R-BAIBA, and (2) their different metabolic source, as evidenced by the AGXT2 polymorphism only affecting R-BAIBA. The proposed function in organ cross talk is supported by the current data and may apply to both enantiomers, but the tissue of origin remains unclear.

## Introduction

β-aminoisobutyric acid (BAIBA), also known as 3-amino-2-methylpropanoic acid, is a non-proteinogenic amino acid. It is a known catabolite of thymine and valine metabolism in mammals. Roberts et al. (2014) recently proposed that, although BAIBA is not a peptide or a protein, it might also be a myokine (Roberts et al., 2014), i.e., “a cytokine or other peptide, produced, expressed and released by muscle fibers and exerting either paracrine or endocrine effects” (Pedersen et al., 2007). First, Roberts and coworkers (Roberts et al., 2014) showed that BAIBA was secreted by myocytes overexpressing the exercise-induced transcription factor PGC-1α. Second, they established that after 3 weeks, plasma BAIBA concentration increased by 20% in mice having access to a running wheel compared to their sedentary littermates. In humans, a chronic elevation of 17% was observed following 20 weeks (3 days/week) of aerobic exercise in previously sedentary and healthy subjects. However, potential acute changes of plasma BAIBA in response to exercise, which is a common characteristic of myokines, were not investigated by these authors at that point. Third, it was suggested that enhanced plasma BAIBA concentrations could have systemic functions. This statement is supported by these and several other investigators who found systemic effects of BAIBA supplementation as reviewed by Tanianskii et al. (2019). For example in mice, supplementation of BAIBA increased the expression of brown adipocyte-specific genes in white adipose tissue and increased the hepatic β-oxidation through a PPARα mediated metabolism (Roberts et al., 2014). Exogenous BAIBA supplementation also led to a decrease in body fat in mice. Finally, the authors suggesting that BAIBA is a myokine found that, in humans, BAIBA levels were inversely correlated to cardiometabolic risk factors (Roberts et al., 2014). Based upon all information available it was therefore hypothesized by different authors that muscle-derived BAIBA could be a mechanistic component of the well-established beneficial effects of physical exercise in chronic metabolic diseases (Ginter and Simko, 2014; Kammoun and Febbraio, 2014). Unfortunately, independent investigators have not yet confirmed this hypothesis in humans. In contrast, Morales et al. (2017) did not find acute changes in plasma BAIBA concentrations following a 350 kcal exercise at 70% of VO_2_peak (Morales et al., 2017). Altogether, there is at this moment no evidence for an acute exercise-mediated effect on plasma BAIBA.

**FIGURE 1:**
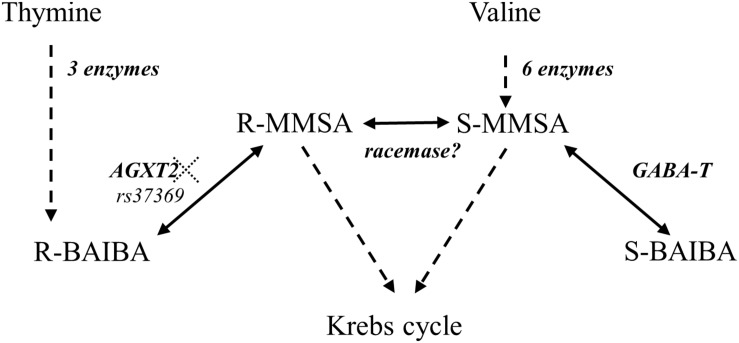
Metabolic pathways involved in R- and S-BAIBA. Dotted lines represent multiple enzymes. AGXT2: alanine glyoxylate aminotransferase 2 (EC 2.6.1.44). GABA-T: 4-aminobutyrate transaminase (EC 2.6.1.19). rs37369 affects AGXT2 activity. Adapted from Van Kuilenburg et al. (2004).

Interestingly, BAIBA has a chiral center and therefore it has two enantiomers: R-BAIBA and S-BAIBA. While very little is known about the physiological role of BAIBA, even less is known about the potential difference in physiological behavior of its enantiomers. R-BAIBA is derived from thymine in three steps that take place in the cytosol of primarily liver and kidney cells (Figure 1; Van Kuilenburg et al., 2006). Subsequently, R-BAIBA can be further metabolized by the transaminase AGXT2 (EC 2.6.1.44) into *R*-methylmalonate semialdehyde (MMSA) in the mitochondria (Rodionov et al., 2014). On the other hand, S-BAIBA is derived from valine in the mitochondria of primarily skeletal muscle (Brosnan and Brosnan, 2006). S-BAIBA is both formed and degraded by GABA-T (EC 2.6.1.19) via the metabolite S-MMSA. Both R- and S-MMSA can be metabolized into propionyl-CoA and further metabolized into the tricarboxylic acid cycle. Although it has been suggested, there is at this point no solid evidence for a spontaneous or enzymatic racemization that might be able to convert R-/S- BAIBA directly or via R-/S- MMSA in healthy humans (Van Gennip et al., 1981; Tamaki et al., 1990; Roe et al., 1998). Despite its known distinct metabolism, the physiological function of the BAIBA enantiomers has not been studied separately, until recently. Kitase et al. (2018) showed that S- and not R-BAIBA was able to protect osteocytes from cell death induced by oxidative stress (Kitase et al., 2018). Additionally S- and not R-BAIBA was secreted from extensor digitorum longus and soleus in a mouse *ex vivo* contractility protocol (Kitase et al., 2018).

In plasma, the concentration of the total amount of BAIBA is reported to be in the low μM range (Van Kuilenburg et al., 2004), whereas urinary concentrations have been reported in a very broad range (Gartler, 1956). The large inter-individual variability of BAIBA in urine relates to the presence of the so-called hyper-BAIBA trait in some individuals. This autosomal recessive trait causes urinary BAIBA levels to increase 100-fold compared to normal concentrations. Hyper-BAIBA excretion has been linked to different single nucleotide polymorphisms (SNPs) in the AGXT2 gene (Nicholson et al., 2011; Seppala et al., 2014; Yoshino et al., 2014) that decimate the enzyme activity (Kittel et al., 2013). The relation between BAIBA and SNP rs37369 has been identified in a genome wide association study as the most deterministic SNP explaining the concentration of BAIBA in urine (Suhre et al., 2011). Additionally, the 1000 genomes project shows that rs37369 TT genotype, associated with low activity, has the highest prevalence of all AGXT2 SNPs with marked difference between African and East Asian populations (>30%) compared to Caucasian (<1%) (The 1000 Genomes Project Consortium, 2015). A conceivable possibility would be that the rs37369 SNP induces altered metabolism and accumulation of only R-BAIBA, as AGXT2 is not involved in the S-BAIBA metabolism. In urine, the amount of R-BAIBA is more than 95% of total BAIBA (Solem et al., 1974), while the relative contribution of R- and S-BAIBA to the total amount of BAIBA in plasma is still in debate. Some authors reported that R-BAIBA was 20% (Solem et al., 1974), while others reported it to be 53% (Gejyo et al., 1976).

The first aim of this study is to elucidate the baseline concentrations and ratio of both enantiomers in plasma and urine. In addition, this study investigates whether or not an exercise-induced plasma increase of R- vs. S-BAIBA occurs. Third, individuals with the TT genotype for SNP rs37369 provide a healthy human AGXT2 knock-down model making it possible to determine whether this enzyme is indeed only involved in the homeostatic control of R-BAIBA, and not S-BAIBA. The latter would provide evidence for separate BAIBA enantiomer metabolism in humans.

## Materials and Methods

### Subjects

In a first phase, 322 Caucasian subjects were genotyped for rs37369. Fifteen healthy recreationally active male (*n* = 12) and female (*n* = 3) subjects (age: 23.5 ± 3.4 year, body mass: 74.6 ± 12.7 kg, height 180.1 ± 7.4 cm) were included in the study. Hereof 3 TT [homozygous for minor allele; associated with low AGXT2 activity (Kittel et al., 2014; The 1000 Genomes Project Consortium, 2015)], 7 CC [homozygous for major allele; associated with normal AGXT2 activity (Kittel et al., 2014; The 1000 Genomes Project Consortium, 2015)] and 5 (heterozygous) participants matched for sex, body mass, body height, and activity/fitness level were recruited. Written informed consent to participate in the randomized cross-over interventional study was obtained from all participants conform the Declaration of Helsinki and the study was approved by the Ghent University Hospital Ethical Committee.

### Screening

DNA was isolated from fresh whole venous blood (EDTA coated vacutainer) with Gentra Puregene Blood Core Kit according to manufacturer’s instructions with minor adaptations (Qiagen, Hilden, Germany), prelevated from an antecubital vein at rest. Incubation time with RBC lysis and cell lysis solution was increased from, respectively, 10 min to 1 h and 0 min to 2 h. Based on the A260/A280 ratio, DNA quality (1.8–2) and DNA concentrations was assessed using the nanodrop 2000C spectrophotometer (Thermo scientific, Wilmington, MA, United States). Rs37369 genotyping using high resolution melting (HRM) was performed based on Sutter et al. (2013). In short, PCR and HRM was carried out on a LightCycler 480 system (Roche, Penzberg, Germany) using a 8.1 μl reaction mix containing 0.5 μl DNA (25 ng.μl^–1^), 0.4 μL primermix (4 μM), 6 μl JumpStart^TM^ Taq Readymix with MgCl_2_ (Merck Life Science, Darmstadt, Germany) and 1.2 μl SYBR Green PCR Master Mix (Applied Biosystems, Foster city, CA, United States). Analysis was performed under the following parameters: pre-incubation: 95°C for 3 min. Amplification/quantification: 30 s at 95°C, 30 s at 58°C and 1 min at 72°C. Melting curve: 1 min at 95°C, 1 min at 40°C, 1 s at 65°C, ramp rate 0.02°C.s^–1^ up to 90°C; 25 acquisitions per °C. Using LightCycler 480 Gene scanning software (vs. 1.5.1) the normalized and shifted melting curves were analyzed.

Forward and reverse primer (CAT TGG AGG GTG GAA GAA GA and CAG AAA GGT GAA TGC AGT GG) were designed using Primer3plus software. Both primers are 20 base pairs in length. The primer melting temperature is respectively, 60.0 and 59.3, whereas the product size is 79 base pairs.

### Study Design – Blood and Urine Sampling

The experiment consisted of three experimental test days. On the first test day, following a medical screening, subjects performed an incremental cycling test (50 Watt for 3 min + 25 Watt.min^–1;^ Lode Excalibur Sport, Groningen, Netherlands) during which gas exchange was measured (breath-by-breath; Jaeger Oxycon Pro, Viasys Healthcare GmbH, Höchberg, Germany). Participants were instructed to keep their cadence between 70 and 80 rpm, and strong verbal encouragement was provided throughout the test to ensure maximum effort. The protocol was terminated at voluntary exhaustion, which was defined as the inability to maintain a minimal cadence of 70 rpm for more than five consecutive seconds. Heart rate (HR) was monitored on a continuous basis (H7 Sensor; Polar, Kempele, Finland). Based upon this incremental test, maximal power output (P_peak_) and VO_2__Peak_ were determined. Breath-by-breath VO_2_ data were transformed into 10-s values for further analysis, and VO_2__Peak_ was defined as the highest 30-s average achieved during the test.

On the other test days, subjects returned fasted and by passive transport to the lab. At arrival, a catheter was inserted in an antecubital vein and subjects were asked to empty their bladder, but urine was not yet collected. Subsequently, during a 20 min period, subjects ate breakfast (ingredients that could be chosen: white bread, jam, chocolate paste, cheese, banana), and kept record of what and how much they ate. Subjects could drink water at libitum. One hour after the start of the breakfast subjects started cycling at 40% of their P_peak_ or remained at rest (test day exercise or rest). Subjects were randomly assigned to first perform either the exercise or the rest test day. At test day “exercise”, subjects were instructed to cycle on an ergometer (Lode Excalibur Sport, Groningen, Netherlands) for 1 h at a cadence of their choice and every 15 min rate of perceived exertion (RPE) was evaluated using a 10 point borg scale. Ten min before the exercise, subjects were instructed to empty their bladder for urine sampling. An aliquot was kept at −20°C. Just before (0’), after 30 and 60 min of cycling and 30 (90’) min following the exercise, blood was obtained via the catheter. Blood was withdrawn using heparin coated vacutainers (Vacutest Kima, Italy), centrifuged at 11000 *g* (centrifuge 5702 R, Eppendorf) and the plasma was stored at −20°C before further analysis. During the whole test subjects were allowed to drink water at libitum. At test day “rest” food intake, blood and urine sampling was kept identical. The first, second and third test day were each separated by at least 1 week.

The exercise intensity of the 1-h exercise was chosen based on evidence that aerobic exercise, such as the 1-h exercise at 40% P_peak_ (Bex et al., 2015), is known to activate PGC1-α (Russell et al., 2003) and that aerobic exercise and PGC1-α are known to modulate BAIBA (Roberts et al., 2014).

### Plasma and Urinary S- and R-BAIBA Determination

Urine and plasma samples (200 μl) were deproteinized by thoroughly mixing with 20 μl of 5 and 35% (w/v) sulphosalicylic acid, respectively, and samples were stored at 4°C for 30 min. After centrifugation (11000 *g*, 10 min), 150 μl of urine and plasma supernatants were collected and mixed with 150 μl of lithium citrate buffer pH 2.2 (Biochrom, United Kingdom). Urine samples were further diluted 1:10 with the same lithium citrate buffer before putting the vials in a Gilson autosampler. S- and R-BAIBA were detected in plasma and urine samples with fluorescence detection of orthophthaldialdehyde-N-isobutyryl-L-cysteine derivatives of S- and R-BAIBA after separation with dual-column reversed-phase HPLC, essentially as described before (Brückner et al., 1995). Thirty five μl OPA (30 mM) – IBLC (45 mM) in a 0.8 M K- borate buffer was added to 70 μl sample by the autosampler, this solution was mixed by three times up and down pipetting, before 80 μl was added to a reaction vial. Following an incubation time of 30 s 50 μl was injected to column I (SUPELCOSIL^TM^ LC-18-DB). Solvent A used for chromatography on column I consisted of 0.17 M Sodium acetate and 16.7% acetonitrile (pH 5), while solvent B consisted of 90.9% Methanol. The compounds of interest eluting from column I were introduced to column II (Altima C18) between minute 14–20 and elution was performed with 0.1 M sodium acetate and 26% acetonitrile (pH 4.8) with a flow of 1 ml.min^–1^. Fluorescence detection was performed using an excitation and emission wavelength of 330 nm and 450 nm, respectively. For plasma, the limit of quantification was 0.02 μM for R- and S-BAIBA. The within-run variability for concentrations similar to those observed in this study was <5%, while the between run variability was about 6% for R-BAIBA and 16% for S-BAIBA. Recovery was 104.9 ± 6.6% R- and 107.0 ± 5.1% for S- BAIBA. For urine, the limit of quantification was 0.05 μM for R- and S-BAIBA. The within-run variability was 7 and 8% for R- and S-BAIBA, respectively, while the between run variability was about 8% for R-BAIBA and 9% for S-BAIBA. Recovery was 99.4 ± 6.2 and 95.7 ± 3.7% for R- and S- BAIBA, respectively.

### Albumin and Creatine Kinase Determination

Albumin and Creatine Kinase (CK) were determined in serum samples using a commercially available Roche Cobas system at a clinical laboratory.

### Statistics

All quantitative variables were tested for normality using a Shapiro–Wilk test. S-BAIBA was normally distributed in plasma and urine. In contrast, circulating and urinary R-BAIBA did not have a normal distribution. Kruskal Wallis test, followed by Mann–Whitney test was performed to compare the difference in baseline values (age; body weight; length; P_peak_; VO_2_peak, R- and S-BAIBA) between the three genotypes. To investigate the effect of exercise on R- and S-BAIBA concentrations in plasma a multivariate 2 × 4 repeated-measures MANOVA was used with “condition” (cycling, rest) and “time” (0′, 30′, 60′, and 90′) as within-subjects factors and R- and S-BAIBA as different measures. Consecutively, pairwise comparisons were used to compare the different time points. When the variable of interest was not normally distributed a Wilcoxon test was performed. In order to investigate the influence of the AGXT2 genotype on plasma R- and S-BAIBA kinetics a multivariate 3 × 2 × 4 repeated-measures ANOVA was used with “genotype” (TT, CT, CC) as a between-subjects factor and “condition” (cycling, rest) and “time” (0′, 30′, 60′, and 90′) as within-subjects factor. Spearman and Pearson correlations were run with variables that were, respectively, normally or not-normally distributed. All statistical analyses were performed using the Statistical Package for the Social Sciences (version 23.0; SPSS, Chicago, IL, United States). Values are presented as mean ± SD and significance was assumed at *P* < 0.05.

## Results

The anthropometric and physical characteristics were not significantly different between the three groups with different AGXT2 genotypes (CC, CT, TT) (Table 1). The mean (± SD) VO_2_peak and P_peak_ were 50.2 ± 10.2 ml/kg/min and 347 ± 74 Watt, respectively. The actual 1-h aerobic exercise test was performed at 139 ± 30 Watt, which corresponded to 62 ± 9% of VO_2_peak and was below each participant’s aerobic threshold, as evaluated with the gas exchange threshold. RPE of the exercise ranged from 3 (moderate) to 5 (hard).

**TABLE 1:** Baseline characteristics.

		**CC**	**CT**	**TT**	**mean**
Total	*n*	7	5	3	15
	Age (year)	22.3 ± 1.1	25.2 ± 5.5	23.7 ± 2.3	23.5 ± 3.4
	Body weight (kg)	74.7 ± 9.6	71.3 ± 10.5	79.7 ± 23.6	74.6 ± 12.7
	Height (cm)	183 ± 7	175 ± 8	182 ± 6	180 ± 7
	VO_2__peak_ (ml.kg^–1^.min^–1^)	51.9 ± 6.1	48.7 ± 16.2	48.5 ± 8.8	50.2 ± 10.2
	40% P_peak_ (Watt)	146 ± 27	127 ± 37	140 ± 26	139 ± 30
Female	*n*	1	1	1	3
	Age (year)	22.8	24.9	21	22.9 ± 1.9
	Body weight (kg)	63.2	58.8	57.4	59.8 ± 3
	Height (cm)	174	171	176	173 ± 3
	VO_2__peak_ (ml.kg^–1^.min^–1^)	38.7	35	48	40.6 ± 6.7
	40% P_peak_ (Watt)	93	80	110	94.3 ± 15
Male	*n*	6	4	2	12
	Age (year)	22.3 ± 1.2	25.2 ± 6.3	25.0 ± 0.3	23.7 ± 3.7
	Body weight (kg)	76.7 ± 8.9	74.4 ± 9.0	90.8 ± 19.2	78.3 ± 11.3
	Height (cm)	184 ± 6	176 ± 9	186 ± 4	182 ± 7
	VO_2__peak_ (ml.kg^–1^.min^–1^)	54.1 ± 2.0	52.2 ± 16.5	48.7 ± 12.4	52.6 ± 9.7
	40% P_peak_ (Watt)	155 ± 15	139 ± 31	155 ± 0	150 ± 20

*Values are presented as mean ± SD, no differences between genotypes.*

### Baseline R- and S-BAIBA

R-BAIBA was by far the most predominant enantiomer in both plasma and urine. The mean concentration of baseline R-BAIBA was 66.7 times higher in plasma (1734 ± 821 nM vs 29.3 ± 7.8 nM) and 334 times higher in urine (35160 ± 47026 μmol/mol creatinine vs 90.4 ± 47.0 μmol/mol creatinine) than S-BAIBA. Plasma S-BAIBA was only 2.03 ± 0.99% of the total amount of BAIBA, whereas urinary S-BAIBA accounted for 1.18 ± 1.43%. The amount of baseline R-BAIBA was not correlated to the amount of baseline S-BAIBA in plasma nor in urine (plasma *r* = −0.333; *p* = 0.225, urine *r* = 0.116; *p* = 0.705). Additionally, there was no intra-individual difference in baseline plasma and urine R- (*p* = 0.588; *p* = 0.334) and S-BAIBA (*p* = 0.356; *p* = 0.881) between test days.

**FIGURE 2:**
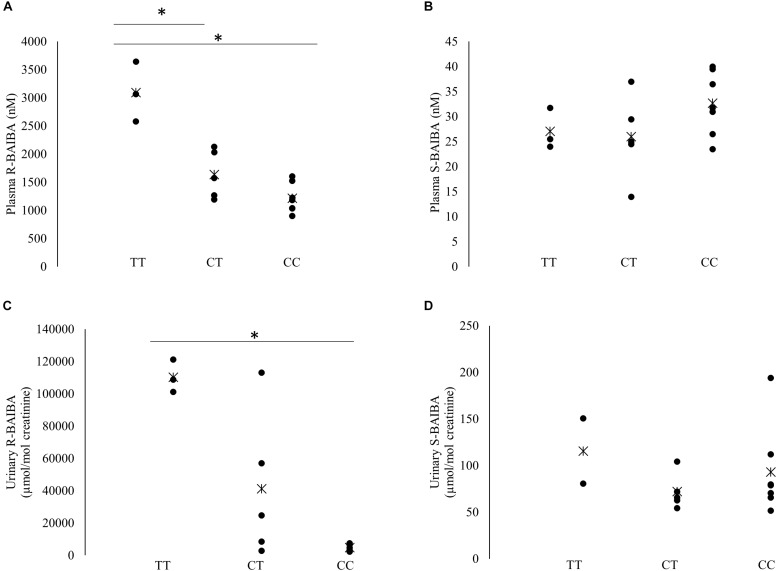
Baseline S- and R-BAIBA concentrations. Individual baseline levels of plasma R- and S-BAIBA **(A,B)** and urinary R- and S-BAIBA **(C,D)** concentrations separated for rs37369 genotype. Cross represents the mean. ^∗^*P* < 0.05.

Baseline plasma R-BAIBA was higher in subjects with TT genotype compared to heterozygous (*p* = 0.025) and homozygous CC (*p* = 0.017) subjects (Figure 2A). R-BAIBA was only trend higher (*p* = 0.062) in subjects with CT compared to CC subjects. In contrast, rs37369 had no effect on plasma S-BAIBA levels (Figure 2B). Similarly, rs37369 strongly affected urinary R-BAIBA (*p* = 0.013), but not S-BAIBA levels (Figures 2C,D). Interestingly, P_peak_ and VO_2__peak_ were positively correlated to baseline concentrations of plasma S-BAIBA (Table 2).

**TABLE 2:** Correlations S-BAIBA.

		**Baseline S-BAIBA**	**Increase of S-BAIBA From 0′–90′**
Age	r	–0.159	–0.354
	p	0.571	0.195
BMI	r	0.150	0.074
	p	0.593	0.794
P_peak_	r	0.745	0.462
	p	0.001^∗^	0.083^[*dollar*]^
VO_2peak_	r	0.760	0.564
	p	0.001^∗^	0.029^∗^

*^∗^*p* < 0.050, ^$^0.05 < *p* < 0.10.*

### Effect of Exercise

**FIGURE 3:**
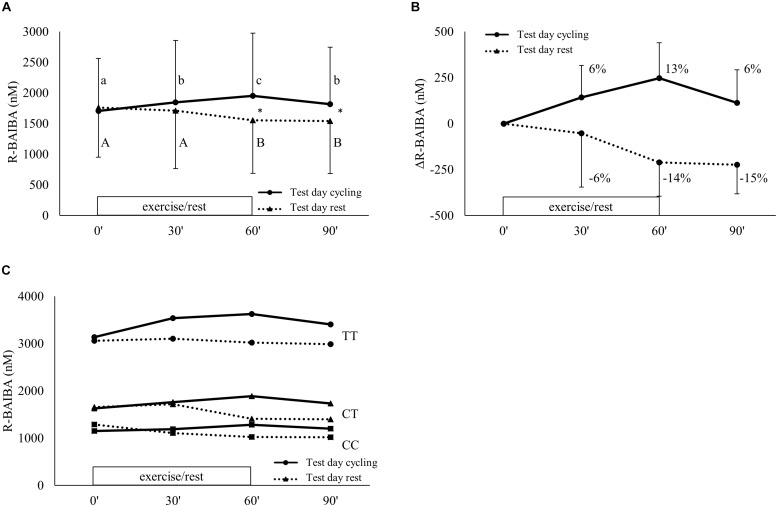
R-BAIBA kinetics during exercise. Plasma R-BAIBA kinetics are shown for **(A)** the actual concentrations, **(B)** the delta change over time and **(C)** the actual concentrations of the different groups based on rs37369 genotype. Full lines represent cycling condition and dotted lines represent resting condition. Exercise was performed from 0 up to 60 min on test day cycling. **(A,B)** show mean ± SD, **(C)** only mean. Different letter indicates a significant difference from other time points during cycling (Arabic) or rest (Arabic capital). ^∗^*P* < 0.05 comparison cycling and rest at same time point. Percentage differences are calculated from baseline levels.

One hour of exercise at 40% of P_peak_ evoked an increase of circulating R-BAIBA compared to remaining in rest (condition^∗^time *p* < 0.001) (Figure 3A). There was an increase after 30 min (+ 6%; *p* = 0.012) and an additional increase after 60 min (+13% compared to baseline, *p* = 0.001) of exercise (Figure 3B). During recovery, R-BAIBA returned to levels similar to levels after 30 min (+6% compared to baseline, *p* = 0.035). On the rest day, a slight decrease in plasma R-BAIBA was observed after 60 and 90 min (*p* = 0.05). R-BAIBA concentrations were significantly higher on the cycling day compared to the rest day at 60 min (*p* < 0.001) and 90 min (*p* = 0.003). In the rs37369 TT genotype, R-BAIBA concentrations were higher (main effect of genotype; *p* < 0.001), but the exercise-induced kinetics were not significantly affected by genotype (condition^∗^time^∗^genotype *p* = 0.503) (Figure 3C).

**FIGURE 4:**
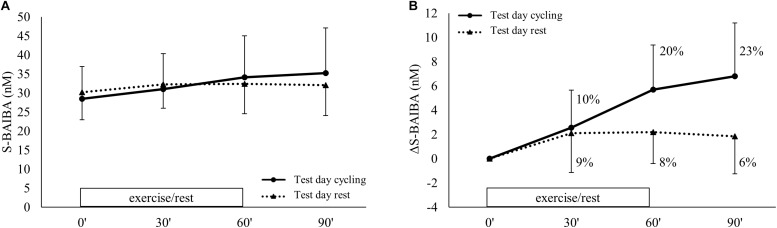
S-BAIBA kinetics during exercise. Plasma S-BAIBA kinetics are shown for **(A)** the actual concentrations and **(B)** the delta change over time. Full lines represent cycling condition and the dotted lines represent resting condition. Exercise was performed from 0 up to 60 min on test day cycling. Figures show mean ± SD. Different letter indicates a significant difference from other time points during cycling (Arabic) or rest (Arabic capital). Percentage differences are calculated from baseline levels.

During exercise, the plasma kinetics of S-BAIBA were significantly different from the rest day (condition^∗^time *p* < 0.001) (Figure 4A). There was an increase of S-BAIBA from baseline to 30 min (+10%; *p* = 0.007) and 60 min of exercise (+20%; *p* < 0.001 compared to baseline), whereafter S-BAIBA concentrations remained elevated during recovery (+23%; *p* < 0.001 compared to baseline) (Figure 4B). At the rest day, there was an increase of 9% after 30 min (*p* = 0.023) that did not change over the following hour. Although there was a significant interaction effect, there was no significant difference between the absolute concentrations of S-BAIBA at any time point between the two conditions. Genotype had no effect on the S-BAIBA kinetics (condition^∗^time^∗^genotype *p* = 0.398; main effect genotype *p* = 0.166). The increase of S-BAIBA after the exercise and the 30 min recovery was significantly positively correlated to VO_2__peak_ (Table 2).

### Markers of Hemoconcentration and Muscle Damage

We measured plasma albumin levels in order to exclude that the observed exercise-induced changes were evoked by hemoconcentration (Alis et al., 2015). Albumin levels remained constant during both rest and exercise days (condition^∗^time: *p* = 0.263) (Table 3). In order to investigate if muscle damage occurred during exercise, the plasma concentration of CK was determined (Brancaccio et al., 2010). During and after cycling CK remained constant suggesting that our intervention did not cause muscle damage (condition^∗^time: *p* = 0.994) (Table 3).

**TABLE 3:** Markers of hemoconcentration and muscle damage before during and following a 1-h aerobic exercise.

	**Cycling**				**Rest**			
		
	**0′**	**30′**	**60′**	**90′**	**0′**	**30′**	**60′**	**90′**
Albumin (g.l^–1^)	49.3 ± 3.3	49.1 ± 5.8	48.3 ± 4.4	47.1 ± 4.4	47.5 ± 3.2	47.5 ± 5.3	46.3 ± 4	47.5 ± 4.7
Creatine kinase (U.l^–1^)	120.5 ± 57.4	119.8 ± 61.1	117.7 ± 57.9	117.5 ± 57.4	99.6 ± 34	99 ± 38.8	96.1 ± 34.4	96.9 ± 31.8

*Mean ± SD are shown.*

## Discussion

A first aim of this study was to elucidate the ratio and homeostatic concentrations of both BAIBA enantiomers. In this study, the plasma concentrations of R-and S-BAIBA ranged from 770 to 4120 nM and from 14 to 61 nM, respectively. This means that “total” BAIBA was about 794 to 4147 nM, which is in agreement with earlier studies (Armstrong, 1963). We found that the total amount of BAIBA in plasma actually consists of 98% R-BAIBA and only 2% S-BAIBA. This is markedly different from the 20% (Solem et al., 1974) and 53% R-BAIBA (Gejyo et al., 1976) that has been suggested earlier. On the other hand, the urinary “total” BAIBA concentrations reported in this study well resembles the concentrations that have been reported previously (Suhre et al., 2011). Also the relative proportion of urinary R-BAIBA is about 99%, which is comparable to earlier reports (Solem et al., 1974). Here we show that the ratio of the enantiomers is not different between plasma and urine, contradicting literature suggesting a difference in renal clearance for R- and S-BAIBA (Solem et al., 1974). It is hard to speculate why there is such high discrepancy in the ratio of enantiomers in plasma between the present and the former studies. Gejyo et al. (1976) suggested that difference in origin (East Asian compared to European) might have been responsible, but this cannot explain the difference between our current result and the investigation of Solem et al. (1974). However, Solem et al. (1974) only used one pooled sample from 2 persons for their experiment. The current method has been successfully used and therefore validated in a clinical setting in the screening and follow-up of patients with pyrimidine degradation defect, which strengthens our confidence in the current method. However, due to current discrepancy in literature, independent confirmation is preferable.

The imbalance between the BAIBA enantiomers suggests that their metabolic pathways and sources could be separate and unrelated. A useful approach in this respect is to explore whether a polymorphism that affects AGXT2 activity would only affect R-BAIBA as can be derived from the proposed metabolic pathways (Figure 1). Indeed, we confirm that a deficient AGXT2 enzyme (TT genotype for rs37369), leading to the hyper-BAIBA trait (Suhre et al., 2011), only affects R-BAIBA but not S-BAIBA levels, in both plasma and urine. Although we recruited only three TT-subjects (due to the very low prevalence of this genotype in our Caucasian population), we believe these conclusions are valid, since the effect size is large and non-parametric statistical analysis was performed to account for the low sample size. Moreover, the CT subjects seemed to be stepwise intermediate to the TT and the CC group for R-BAIBA, but not S-BAIBA. Therefore, the current results indicate that in healthy humans the metabolic pathways of R- and S-BAIBA are most likely unrelated, and that they should be investigated as separate physiological entities.

The second aim of this study was to elucidate whether or not an acute exercise response of R- and S-BAIBA exists. Following 30 min of cycling, an increase of R-BAIBA was already observed. This increase is, although moderate, significant. Not only R-BAIBA, but also circulating S-BAIBA gradually increased during exercise by about 20%. Although this absolute and relative increase is small, it is statistically significant and suggests an exercise-related metabolism. We demonstrate that the acute changes observed in this investigation reflect true changes and are not caused by exercise-induced hemoconcentration, as illustrated by the albumin measurements.

Only two investigations have been published about the effect of exercise on BAIBA levels in humans (Roberts et al., 2014; Morales et al., 2017). The percentage changes in “total” BAIBA measured in this investigation are in the same order of magnitude as was observed by the chronic intervention in mice and humans reported by Roberts et al. (2014). Unfortunately, these authors did not report the absolute concentrations making it difficult to compare with the current investigation. Additionally, it is unknown whether the 17% increase of “total” BAIBA, was primarily caused by R-BAIBA or S-BAIBA. We now demonstrate that R-BAIBA is responsible for 97.8% of the 252 nM increase in total BAIBA after 60 min acute exercise, and it could be hypothesized that the same holds true for the observed chronic effects (Roberts et al., 2014). Our results are not in congruence with Morales et al. (2017) who did not find an increase of BAIBA following a 350 kcal acute exercise at 70% VO_2_peak, lasting about 30 min. This difference in results could be due to differences in study design. In contrast to our study, they did not perform a crossover design where the subjects did not exercise. Additionally, we observed the highest effects after 60 min of exercise, whereas their exercise had a shorter duration.

The current investigation does not provide evidence for the origin of R- and S-BAIBA in the circulation. Either this can be released from an endogenous storage or it could be acutely formed. Based on the current literature it cannot be excluded that part of the increase is caused by previously stored R- or S-BAIBA. “Total” BAIBA has been detected, but not quantified in mice (Roberts et al., 2014; Hatazawa et al., 2015) and human (Fazelzadeh et al., 2016) muscle, but information of the single enantiomers is lacking. On the other hand, there is evidence toward acute production of both enantiomers as well. The acute increase of R-BAIBA could possibly be explained by a higher pyrimidine turnover during exercise, although this suggestion is based upon measurement of only uridine and not thymine (Yamamoto et al., 1997). Since there is only limited evidence for thymine degradation during exercise at present, R-BAIBA might be used as a marker as proposed by Solem et al. (1974). Interestingly, acutely formed R-BAIBA is most likely not muscle derived as thymine degradation activity is very low in muscle compared to liver and kidney (Van Kuilenburg et al., 2006), implying that acutely formed R-BAIBA might be an exerkine, an exercise induced factor released from any organ during exercise, rather than from muscle (alone). This hypothesis is supported by the findings of Kitase et al. (2018) who did not find increased levels of R-BAIBA after contraction of isolated muscle in mice (Kitase et al., 2018). In contrast, these investigators did find an increase of S-BAIBA in isolated muscle.

S-BAIBA might also be acutely formed as the degradation of valine, a BCAA and precursor of S-BAIBA (Figure 1), has been shown to take place in muscle and liver during acute exercise (Brosnan and Brosnan, 2006). We also observed an additional increase of S-BAIBA following exercise suggesting that BCAA degradation occurs during recovery. Training status might also improve BCAA degradation capacity (Mckenzie et al., 2000; Hatazawa et al., 2014). Indeed, we could observe a significant positive correlation between VO_2_peak and both baseline S-BAIBA and the exercise-induced increase of plasma S-BAIBA. This correlation is not surprising as PGC-1α has been shown to increase BAIBA in muscle (Roberts et al., 2014; Hatazawa et al., 2015). Most likely, PGC-1α, as a transcriptional co-activator, does not affect BAIBA levels acutely and directly, but rather in a chronic fashion, through mitochondrial biogenesis as both GABA-T (S-BAIBA) and AGXT2 (R-BAIBA) are mitochondrial enzymes. Additionally, the entire valine degradation pathway is mitochondrial which might further affect the formation of S-BAIBA. How PGC-1α could impact thymine degradation and therefore R-BAIBA, is unclear at this point. Altogether, the results indicate that although the metabolite S-BAIBA is not a peptide, it might indeed have myokine-like functions. As CK levels, the common marker of muscle damage, remained constant we consider it to be an active release, and not a leakage through muscle damage.

The expression of both mitochondrial enzymes GABA-T and AGXT2 (Figure 1), necessary to produce, respectively, S- and R-BAIBA are quite low in muscle and high in liver. Additionally, muscle have minimal thymine degradation activity, but have the ability to degrade valine, while liver is able to degrade both. Finally, Kitase et al. (2018) only found an increase of S-BAIBA from muscle. Therefore, the liver might be a promising target for further research with respect to the endogenous source of BAIBA’s enantiomers by exercise. Additionally, dipeptides containing BAIBA have been measured in mammalian tissue (Morris et al., 1961; Kakimoto et al., 1965) which could also be relevant to look at, especially since the metabolically linked β-alanine is mainly stored in muscle in conjugation with histidine (β-alanyl-L-histidine, also called carnosine) (Boldyrev et al., 2013).

It is hard to speculate on the physiological meaning and relevance of the observed changes in R- and S-BAIBA. Only Kitase et al. (2018) looked at the physiological roles of both R- and S-BAIBA separately, indicating that S-BAIBA and not R-BAIBA is a bone-protecting factor (Kitase et al., 2018). The 13% (R) and 20% (S) increase, measured in the current investigation, is in the same relative range as reported for other myokines or exerkines such as irisin [15% (Fox et al., 2018)] and BDNF [13–30% (Ferris et al., 2007)], but lower than for example the 100-fold changes measured in the IL-6 (Fischer, 2006). Additionally, at baseline, these myokines are present at similar or even lower concentrations than S-BAIBA. This indicates that although the baseline concentrations and the changes might be considered low, they could be relevant. Further research needs to focus on the functional properties of both enantiomers. Additionally, the determinants (e.g., training and nutritional status, exercise intensity,.) of the release of both BAIBA enantiomers and the minimal effective changes necessary to induce physiologically relevant effects are unclear. Altogether, it is too early to talk about possible paracrine or endocrine effects of BAIBA, as such.

The most important limitation of this study is the small samples size, due to the low incidence of the TT-genotype in Caucasians, generating a risk of false positive or negative results. This should be taken into consideration when interpreting the results presented in this manuscript. However, by using a homogenous sample, matched groups and a crossover design, together with the fact that homogenous effects were measured, the results are certainly relevant. As the participants were Caucasian, physically active, healthy and performed only exercise on one intensity, generalization of results presented here warrants caution until further confirmation.

## Conclusion

In conclusion, this study first showed that BAIBA predominantly consists of R-BAIBA (±98%), both in plasma and urine. Plasma and urinary R-BAIBA, but not S-BAIBA, is markedly affected by the AGXT2 rs37369 TT genotype underlying the hyper-BAIBA trait. This is one of several indications that R- and S-BAIBA have separate sources in humans. In relation to the previously suggested role of BAIBA as a myokine, we demonstrated that plasma levels of both R- and S-BAIBA are moderately but significantly elevated (13–20%) by acute aerobic exercise in humans. The physiological effects, for example in organ crosstalk or the source tissue (myokine or exerkine) of each enantiomer warrants further investigation.

## Data Availability Statement

The datasets generated for this study are available on request to the corresponding author.

## Ethics Statement

Written informed consent to participate in the randomized cross-over interventional study was obtained from all participants conform the Declaration of Helsinki and the study was approved by the Ghent University Hospital Ethical Committee.

## Author Contributions

JS, IE, LB, and WD conceived and designed the experiment. JS, FL, IE, and LB performed the experiment. AV, LS, and FV analyzed the samples. JS, IE, LB, and WD interpreted the results. All authors wrote and approved the final version of the manuscript.

## Conflict of Interest

The authors declare that the research was conducted in the absence of any commercial or financial relationships that could be construed as a potential conflict of interest. The reviewer SB and handling Editor declared their shared affiliation at the time of the review.

## Abbreviations

AGXT2: alanine - glyoxylate aminotransferase 2
BAIBA: β-aminoisobutyric acid
BCAA: brached chain amino acids
GABA-T: 4-aminobutyrate-2-oxoglutarate transaminase
MMSA: methylmalonate semialdehyde.
